# Classification of Multiple H&E Images via an Ensemble Computational Scheme

**DOI:** 10.3390/e26010034

**Published:** 2023-12-28

**Authors:** Leonardo H. da Costa Longo, Guilherme F. Roberto, Thaína A. A. Tosta, Paulo R. de Faria, Adriano M. Loyola, Sérgio V. Cardoso, Adriano B. Silva, Marcelo Z. do Nascimento, Leandro A. Neves

**Affiliations:** 1 Department of Computer Science and Statistics (DCCE), São Paulo State University (UNESP), Rua Cristóvão Colombo, 2265, São José do Rio Preto 15054-000, SP, Brazil; 2 Department of Informatics Engineering, Faculty of Engineering, University of Porto, Dr. Roberto Frias, sn, 4200-465 Porto, Portugal; guilhermefroberto@gmail.com; 3 Science and Technology Institute, Federal University of São Paulo (UNIFESP), Avenida Cesare Mansueto Giulio Lattes, 1201, São José dos Campos 12247-014, SP, Brazil; tosta.thaina@unifesp.br; 4 Department of Histology and Morphology, Institute of Biomedical Science, Federal University of Uberlândia (UFU), Av. Amazonas, S/N, Uberlândia 38405-320, MG, Brazil; paulorfaria1976@gmail.com; 5 Area of Oral Pathology, School of Dentistry, Federal University of Uberlândia (UFU), R. Ceará—Umuarama, Uberlândia 38402-018, MG, Brazil; loyolaam@gmail.com (A.M.L.);; 6 Faculty of Computer Science (FACOM), Federal University of Uberlândia (UFU), Avenida João Naves de Ávila 2121, Bl.B, Uberlândia 38400-902, MG, Brazil

**Keywords:** classification, histological images, deep-learned features, fractal techniques, xAI representation, ensembles, heterogeneous classifiers

## Abstract

In this work, a computational scheme is proposed to identify the main combinations of handcrafted descriptors and deep-learned features capable of classifying histological images stained with hematoxylin and eosin. The handcrafted descriptors were those representatives of multiscale and multidimensional fractal techniques (fractal dimension, lacunarity and percolation) applied to quantify the histological images with the corresponding representations via explainable artificial intelligence (xAI) approaches. The deep-learned features were obtained from different convolutional neural networks (DenseNet-121, EfficientNet-b2, Inception-V3, ResNet-50 and VGG-19). The descriptors were investigated through different associations. The most relevant combinations, defined through a ranking algorithm, were analyzed via a heterogeneous ensemble of classifiers with the support vector machine, naive Bayes, random forest and K-nearest neighbors algorithms. The proposed scheme was applied to histological samples representative of breast cancer, colorectal cancer, oral dysplasia and liver tissue. The best results were accuracy rates of 94.83% to 100%, with the identification of pattern ensembles for classifying multiple histological images. The computational scheme indicated solutions exploring a reduced number of features (a maximum of 25 descriptors) and with better performance values than those observed in the literature. The presented information in this study is useful to complement and improve the development of computer-aided diagnosis focused on histological images.

## 1. Introduction

Histopathology considers the study of biological tissues and their microscopic structures. Histopathologists examine tissue samples under a microscope to identify abnormal changes in cell structure and indicate possible pathological conditions [1,2]. In this process, staining techniques, such as hematoxylin and eosin (H&E), and microscopy are commonly explored in order to obtain information for the diagnosis, treatment and understanding of the progression of hyperplasias, dysplasias, metaplasias and neoplasms [1,3,4]. For example, when the H&E technique is used, the staining process aims to highlight the basophils and eosinophils in tissues [5,6]. The first dye highlights cell nuclei with a bluish color, while the second makes the cytoplasm reddish. Other cellular structures have colors derived from mixing these dyes [7]. These conditions contribute to the analysis of regions of interest, whether by specialists or automated systems aimed at classifying and recognizing cancer patterns, considered a global public health issue. Over the past decade, there has been a 20% increase in cancer incidence, and more than 25 million new cases are expected by 2030. According to [8], cancer has become an important cause of premature mortality globally and is associated with high social and economic costs. The estimated productivity losses are EUR 104.6 billion of the gross national domestic product in Europe and USD 46.3 billion of the combined gross domestic product of the BRICS countries (Brazil, Russia, India, China and South Africa).

In this context, delays in the diagnosis and treatment of cancer can increase the rates of the disease in advanced stages and consequently, mortality. On the other hand, the characterization of cellular changes and their associations with cancer are complex and challenging processes for histopathologists [3,4,9]. Methods based on computer vision and artificial intelligence techniques have provided important advances to minimize these challenges, increasing cancer diagnostic and prognostic accuracy values, especially through computer-aided diagnosis (CAD) [10,11]. In these systems, the extraction and classification of features are essential for the recognition of histological patterns commonly examined by pathologists, especially in the contexts of colorectal cancer, breast cancer, oral dysplasia and liver tissue [12,13]. It is noted, for example, that the combined use of techniques (ensemble learning), such as those based on convolutional neural networks (CNN), make it possible to characterize information at different levels and scales to verify how they relate to each other in the data space [14].

Although a CNN architecture directly performs image classifications, when the values in its internal layers (deep-learned features) were investigated separately, the results indicated more relevant performance, with new approaches in the context of medical images [15,16,17,18,19,20], including the use of transfer learning [21] or even different ensembles of descriptors [22,23,24,25,26,27]. Among the combinations with deep-learned features, the use of fractal descriptors (handcrafted), such as fractal dimension, lacunarity and percolation, deserves to be highlighted because they are capable of appropriately measuring complex shapes generally found in nature and in the context of H&E samples [12,28,29,30,31,32,33,34]. Moreover, the handcrafted features that we selected are commonly used for describing complex structures like the ones found in histopathological images. Therefore, it is noted that proposals based on ensemble learning have been indicated as one of the main research fields for the development of new models [35].

In addition, it is important to note that explainable artificial intelligence (xAI) has contributed significantly to the improvement of ensemble learning models, especially in the validation and interpretation of results [36,37], in order to ensure that accurate classifications are determined for the right reasons [38,39]. This question motivated the development of strategies based on class activation mappings (CAMs) [40], specifically, gradient-weighted class activation mappings (Grad-CAMs) and local interpretable model-agnostic explanations (LIME) [41]. These approaches can be applied to produce visualizations of the image regions that support the CNN classification process. Quantifying this type of image with fractal techniques can complement the process of classification and pattern recognition of histological images. Despite these observations, it is noted that this hypothesis has not yet been investigated in the specialized literature in the context of multiple H&E datasets such as those explored here.

The strategies highlighted previously result in a highly complex feature space, a fact that can make investigations of contexts with a reduced number of samples unfeasible [42,43], such as histological datasets commonly used to investigate colorectal cancer, breast cancer, oral dysplasia and liver tissues. This situation can be overcome by identifying the most relevant features for the classification process and, consequently, indicating more accurate and robust CAD systems. Thus, feature selection plays a critical role in identifying patterns [44], but there is no universal approach in order to define the best results for all contexts [45,46,47,48]. On the other hand, algorithms based on ranking and filters, such as ReliefF, are capable of detecting feature dependencies and provide the best solutions in different experiments [47,49,50,51]. This strategy provides sets of features with different dimensions via any desired criteria. In addition, a set of descriptors can also be highly dependent on the heuristics of the classifier used to evaluate the model [52]. This challenge can be minimized through a classification process with different heuristics (ensemble of classifiers) [53]. Techniques that are part of this set consider the so-called crowd wisdom, in which a decision is made from different perspectives and when associated, can be more accurate. The justification is that possible individual errors are compensated by the successes of the other components [53]. Therefore, investigating the most relevant combinations of descriptors and techniques for the analysis, classification and pattern recognition of H&E images remains an ongoing challenge. This makes ensemble-learning-based solutions more generalizable and robust.

Even with some initiatives observed [12,54,55,56,57], models based on ensemble learning with multiscale and multidimensional fractal descriptors, such as those investigated here, have not yet been fully explored in the literature, including the quantification with the Grad-CAM and LIME representations. In this context, some insights are still pertinent, such as whether it is possible to define standards between the techniques used to classify multiple types of H&E images; whether multiscale and multidimensional fractal descriptors indicate gains in relation to the results achieved via deep-learned features with transfer learning; whether fractal descriptors obtained from Grad-CAM and LIME representations can contribute to the performance of an ensemble learning scheme; and whether the combination of ensembles (descriptors and classifiers) indicates more competitive performance in relation to that available in the specialized literature. Some architectures such as DenseNet-121 [58], Inception-V3 [59], ResNet-50 [60] and VGG-19 [61] can still be explored to provide deep-learned features, even with the success provided in the direct classification of some types of histological images [23,62,63,64]. EfficientNet [65] has not yet been fully explored with the approaches presented here. Therefore, the strategies and conditions previously presented are useful to make knowledge comprehensible to the specialists focused on developing and improving CAD systems. Moreover, the proposed scheme to identify, select and classify the main combinations is based on aspects widely discussed in information theory, image processing and pattern recognition, especially to obtain more robust baseline schemes in this context of histological samples.

In this work, a computational scheme was defined to identify the most relevant feature ensembles in order to complement and improve the development of CAD systems focused on H&E images. The handcrafted descriptors were defined using multiscale and multidimensional fractal techniques (percolation, fractal dimension and lacunarity) to quantify the original H&E samples and the corresponding LIME and Grad-CAM representations. The deep-learned features were obtained from the DenseNet-121, EfficientNet-b2, Inception-V3, ResNet-50 and VGG-19 architectures. The descriptors were analyzed based on different ensembles, considering the ReliefF algorithm with an ensemble of classifiers (support vector machine, naive Bayes, random forest and K-nearest neighbors). The proposed methodology was applied to distinguish histological samples representative of breast cancer, colorectal cancer, oral dysplasia and liver tissue. The information and conditions obtained were detailed from each experiment. The main contributions of this work are:

- A computational scheme capable of indicating the main ensembles of descriptors for the study of histological images, exploring the ReliefF algorithm and multiple classifiers;
- An optimized ensemble of deep-learned features with the best results for classifying colorectal cancer, liver tissue and oral dysplasia, using a reduced number of features (up to 25 descriptors);
- Indications of the discriminative power of ensembles based on fractal features from the LIME and CAM representations;
- Solutions without overfitting and a more robust baseline scheme, with the necessary details for comparisons and improvements of CAD systems focused on H&E images.

In the Section 2 of this paper, the methodology is described in detail, and in Section 3, the results are presented and discussed. The conclusion is drawn in Section 4.

## 2. Materials and Methods

**Figure 1:**
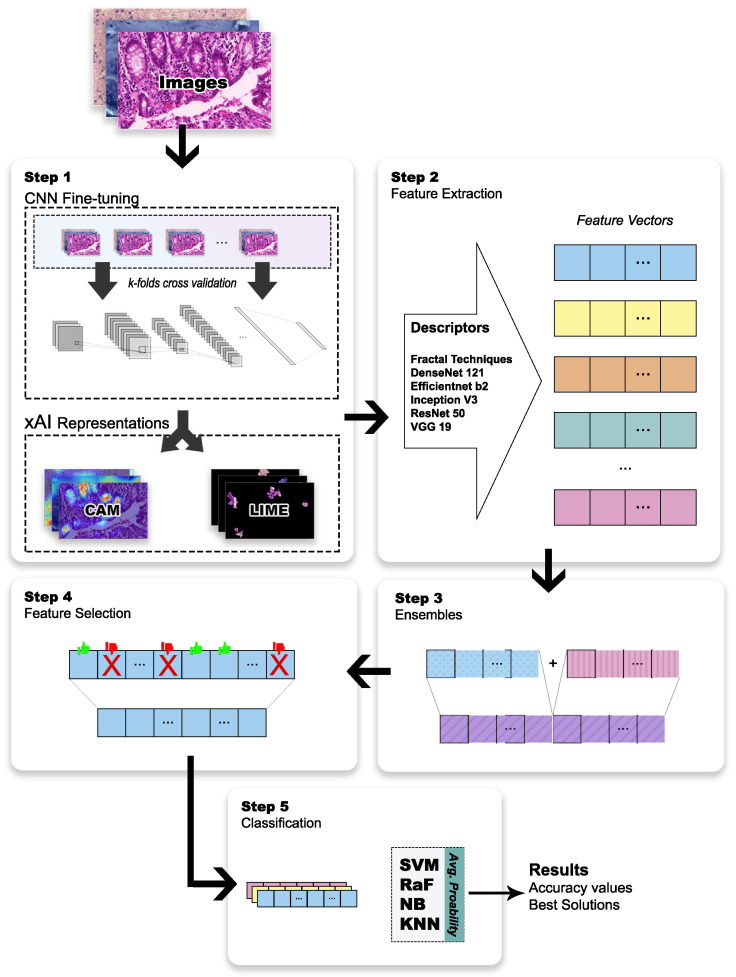
An overview of the proposed scheme.

In this section, the main steps for the proposed scheme are described, exploring the combined use of deep-learned features via transfer learning with fractal descriptors obtained from original H&E images and their CAM and LIME representations. In the first step, the CNN architectures were defined, and the output layer was fine-tuned to match the classes available on each dataset. Also, in the same step, the CAM and LIME representations were generated considering the fine-tuned models. The second step defined the extraction of the deep-learned features from the selected architectures and the multiscale and multidimensional fractal features using the original H&E images and their xAI representations. In the third step, the extracted features were combined (feature vectors) in different ensembles in order to identify the most relevant information in each context. In the fourth step, the features from each ensemble were ranked and selected to define the best solutions with a reduced number of descriptors. In the fifth and last step, the discriminative capacities of the optimized ensembles were verified through a heterogeneous ensemble with four classifiers. An overview of the proposed scheme is illustrated in Figure 1, with details presented in the next sections.

### 2.1. Datasets

**Figure 2:**
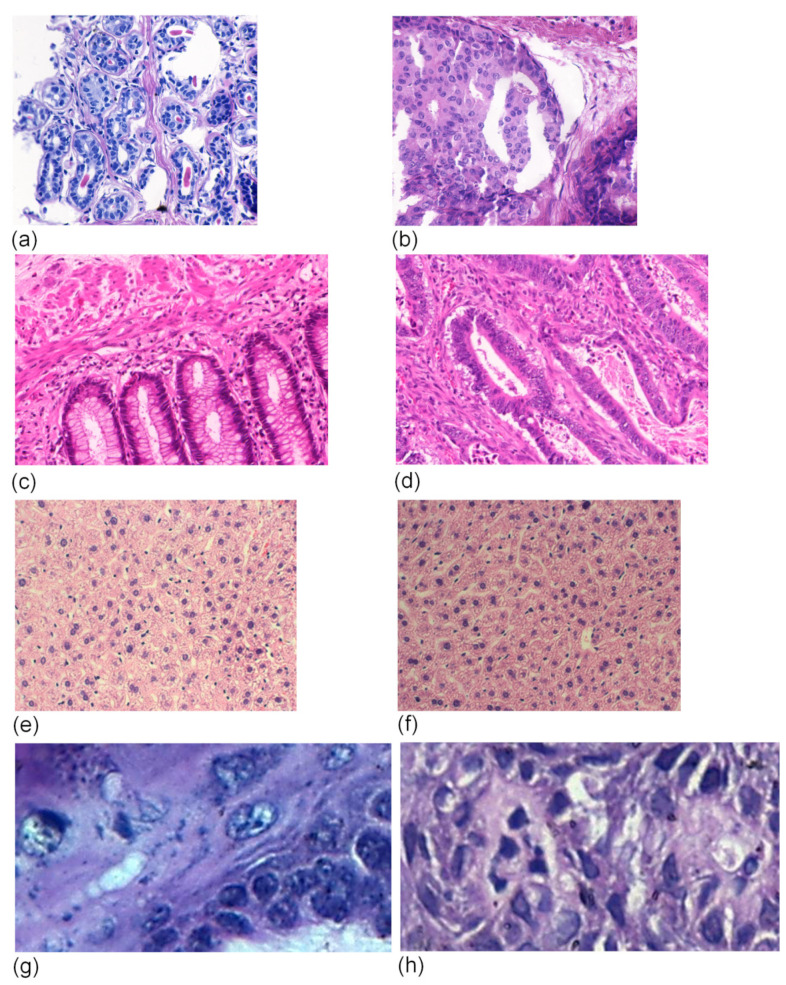
Examples of H&E images: breast UCSB from [66], benign (**a**) and malignant (**b**); CR from [67], benign (**c**) and malignant (**d**); LG from [68], male (**e**) and female (**f**); OED from [69], healthy (**g**) and severe (**h**).

The proposed scheme was tested on four H&E-stained histological datasets, representatives of breast cancer (UCSB) from [66], colorectal cancer (CR) from [67], liver tissue (LG) from [68] and oral epithelial dysplasia (OED) from [69]. The main details about these datasets are in Table 1, with some samples presented in Figure 2 in order to illustrate each context.

### 2.2. Step 1—Fine-Tuning the CNN and xAI Representations

Five CNN architectures were considered in the present scheme: DenseNet-121 [58], EfficientNet-b2 [65], Inception-V3 [59], ResNet-50 [60] and VGG-19 [61]. These models were chosen considering their different image classification strategies, which provided a broader investigation of the proposed scheme with different deep-learned features. All models were obtained from the PyTorch library, with details presented in Table 2, including accuracy values achieved on the ImageNet dataset [70].

The fine-tuning step was applied to map the last layer of each CNN with the available classes, changing the final connections and weights corresponding to the total number of groups in each H&E dataset (Table 1). This strategy avoided the full network training stage and made it possible to investigate datasets with a reduced number of images. This process was performed with a k-fold cross-validation (k=10), which divided the input in *k* folds of approximately the same size in order to train the model from several possible samples. For each of the *k* iterations of the training process, a fold was used as evaluation data and the other k−1 as training data. Each iteration should train a new model, and the output selected was the model with the highest accuracy when classifying test data left out of the process’s training input. This technique was applied to reduce the possibility of overfitting.

In addition, each training considered 10 epochs, using the stochastic gradient descent (SGDM) strategy, and an initial learning rate lr=0.01 with a reduction factor of 0.75 every 2 epochs; the cross-entropy function was used to calculate the adjustment on the parameters. This was repeated for each permutation of architecture and dataset. It is important to highlight that the input images were normalized considering the standard deviation and average of the ImageNet dataset’s color channel values to match the methodology used on the model’s pretraining [72]. Finally, the resulting fine-tuned models were the ones that achieved the highest accuracy for the evaluation set, independent of the epoch.

#### xAI Representations: LIME and Grad-CAM

**Figure 3:**
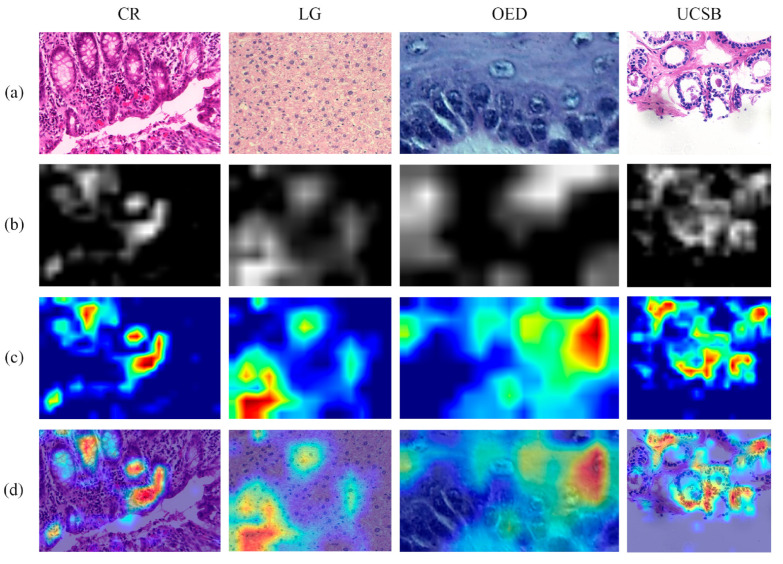
Examples of xAI representations based on the Grad-CAM technique, with (**a**) the original image, (**b**) the weights mapped to indicate the contribution of each pixel, (**c**) the mapping transformed into a heatmap and (**d**) the heatmap overlaying the original image.

To obtain each xAI representation, LIME and Grad-CAM methods were applied to every image using the fine-tuned model for the corresponding dataset. The Grad-CAM representations were defined through the last convolutional layer of each CNN architecture, using the approach of [73]. This choice was based on the idea that the deepest layers contained values related to global patterns on the input [74]. The output was a map, converted into a heatmap, with the weights indicating the contribution of each pixel to the final classification. In Figure 3, the Grad-CAM representations of some H&E images are illustrated.

**Figure 4:**
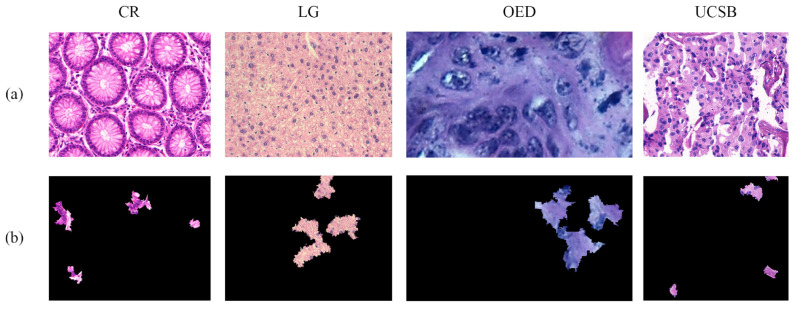
Examples of LIME representations with (**a**) indicating the original image and (**b**) the five selected superpixels for the explanation.

For the LIME method, the results were defined via 1000 local disturbances and using the quick-shift segmentation algorithm [75]. The obtained representation indicated an image with five regions of interest, or superpixels, that were most relevant to explaining the classification result. In Figure 4, some examples of LIME representations obtained from H&E images are illustrated.

### 2.3. Step 2—Feature Extraction

In this study, the attributes were defined from three origins to compose the ensembles: fractal features from the H&E images; deep-learned features from the layer preceding the output on multiple CNN models; and fractal features from the LIME and CAM representations. These three groups were identified as handcrafted features, deep-learned features and xAI features, respectively.

#### 2.3.1. Handcrafted Features: Multiscale and Multidimensional Fractal Techniques

The quantification was carried out using multiscale and multidimensional fractal techniques, specifically the fractal dimension, lacunarity and percolation approaches. The fractal dimension was based on the idea of expanding Euclidean concepts, in which measurements were contained in an *n*-dimensional space, with *n* being an integer greater than zero. The fractal dimension quantified the amount of space filled, indicating the roughness of the structure under analysis. Lacunarity is a complementary measure to the fractal dimension, quantifying the distribution and organization of pixels contained in an image. The lacunarity values represented how the patterns were organized at different observation scales. Percolation is a physical concept that can be observed in the movement and filtering of fluids through porous materials. A classic example of this phenomenon is water flowing through a glass of coffee powder. Considering the quantification process, this concept was explored to indicate the number of clusters, image porosity and cluster size [32].

Fractal techniques were calculated from probability matrices, responsible for storing the probabilities of an image containing a square region filled with each of the shapes that constituted it. This filling was verified via a distance relationship between pixel values under analysis and the size of the area in question [76]. Each matrix was obtained using the *gliding-box* method, which consisted of sliding a square box of side *r* across the entire image and checking whether the pixels were inside or outside the box [77]. Thus, given an *r*-sided box with a central pixel $p_{c}$, a pixel *p* was considered when its distance *d* in relation to $p_{c}$ was less than or equal to *r*. This process resulted in a frequency distribution matrix N(m,r), with *m* representing the number of pixels within a box of side *r*. The probability matrix P(m,r) was obtained through the normalization of N(m,r), according to Equation (1), dividing the value of each count by $n_{r}$, which represented the total number of boxes of side *r* contained in the image with the application of the *gliding box*, as seen in Equation (2).

(1) $$P\left(m,r\right)=\frac{N(m,r)}{n_{r}},$$

(2) $$n_{r}=\left(width-r+1\right)*\left(height-r+1\right).$$

The previously described strategy indicated a multiscale quantification due to the variation in *r*. In addition, as described by [78], in the proposed scheme, the Chebyshev distance or chessboard was applied to calculate *d*. Finally, as the application context uses colored images with RGB color space, the multidimensional strategy was applied considering a pixel via a 5-dimensional representation, such as (x,y,r,g,b) [78].

From these procedures, the fractal dimension FD was calculated considering the estimate of boxes of side *r* necessary to overlay an image, as shown in Equation (3). The lacunarity *L* was calculated from the first and second moments of the probability matrix, obtained according to Equations (4) and (5), respectively. These moments were combined using Equation (6) and resulted in the measurement of the lacunarity on a specific observation scale.

(3) $$FD\left(r\right)=\sum_{m=1}^{r^{2}}\frac{P(m,r)}{m}.$$

(4) $$\mu \left(r\right)=\sum_{m=1}^{r^{2}}mP\left(m,r\right).$$

(5) $$\mu^{2}\left(r\right)=\sum_{m=1}^{r^{2}}m^{2}P\left(m,r\right).$$

(6) $$L\left(r\right)=\frac{\mu^{2}\left(r\right)-{\left(\mu \left(r\right)\right)}^{2}}{{\left(\mu \left(r\right)\right)}^{2}}.$$

Percolation measurements were also extracted using the *gliding-box* algorithm, following the strategy of [32]. Therefore, given a box with side *r*, the pixels contained in the box were considered pores and labeled using the Hoshen–Kopelman [79] algorithm. Pores with the same label were understood as part of the same cluster. This process was repeated for each box of side *r*. Thus, it was possible to compute the metrics C(r), which indicated the average number of clusters present in each box; Q(r), which defined the average coverage ratio of the largest cluster; and P(r), which provided the ratio of percolating boxes.

The C(r) metric was obtained via an average count of the number of clusters *c* (present in each box *i*), according to Equation (7). The metric Q(r) was obtained considering an average of the size of the largest cluster $|c_{max}|$ in each of the *i* boxes, according to Equation (8). Finally, the ratio of percolating boxes was defined by dividing the number of boxes in which percolation occurred by the number of boxes computed in an image. Percolation in a box $p_{i}$ occurred when the ratio between the number of pores $\Omega_{i}$ and the total number of pixels in the box $r^{2}$ exceeded the percolation threshold 0.59275 [12,32], as shown in Equation (9). Thus, P(r) was defined via Equation (10).

(7) $$C\left(r\right)=\frac{\sum_{i=1}^{n_{r}}c_{i}}{n_{r}}.$$

(8) $$Q\left(r\right)=\frac{\sum_{i=1}^{n_{r}}\left|c_{{max}_{i}}\right|}{n_{r}}.$$

(9) $$p_{i}=\left\{\begin{array}{c} 1,\;\frac{\Omega_{i}}{r^{2}}\geq 0.59275,\\ 0,\;\frac{\Omega_{i}}{r^{2}}<0.59275. \end{array}\right.$$

(10) $$P\left(r\right)=\frac{\sum_{i=1}^{n_{r}}p_{i}}{n_{r}}.$$

It is important to highlight that the quantification carried out in this work used matrices defined from boxes with side *r*, within the 3≤r≤41 range, according to descriptions presented by [12,78]. Also, the *r* parameter was set to an odd value to guarantee the existence of a central pixel in each box. Therefore, the increase in the value of *r* was of two units in each iteration. The probability matrix based on these parameters guaranteed a quantification on 20 different scales.

In addition, both lacunarity functions and percolation measures were also interpreted as scalar values in order to obtain representative descriptors of possible patterns existing in each observation [12,32,78,80,81,82,83]. In these proposals, the authors were able to point out how some of these curves displayed a distinct behavior for each of the classes, making them relevant to the classification process. These features were defined as:

- **Area under the curve (A):** It indicates the complexity of the texture. For a discrete function consisting of *N* points defined in $x_{1},\ldots ,x_{n}$, this descriptor can be obtained via Equation (11), with *a* and *b* as the point indices that delimit the analysis range;
  (11) $$A\left(a,b\right)=\frac{b-a}{2N}\sum_{n=a}^{b-1}\left(f\left(x_{n}\right)+f\left(x_{n+1}\right)\right).$$
- **Skewness (S):** it is defined via Equation (12), where *N* is the number of points in the function, $x_{i}$ is the *i*-th point in the function, $\bar{x}$ is the average of the function values, and *a* and *b* are the indices of the points that delimit the interval;
  (12) $$S\left(a,b\right)=\frac{\frac{1}{N}\sum_{i=a}^{b}{\left(x_{i}-\bar{x}\right)}^{3}}{\sqrt{{\left[\frac{1}{N}\sum_{i=a}^{b}{\left(x_{i}-\bar{x}\right)}^{2}\right]}^{3}}}.$$
- **Area ratio (R):** From the asymmetry, the ratio between the halves of the area under the curve must also present similar values for similar classes. This descriptor was obtained through Equation (11), with *a* and *b* indicating the points that delimit the interval;
  (13) $$R\left(a,b\right)=\frac{A(\frac{b}{2}+1,b)}{A(a,\frac{b}{2})}.$$
- **Maximum point:** It indicates the value in the largest heterogeneous area of the curve. Thus, images from the same class can present similar values, for both f(x) and *x*. Totally different values are expected for different classes.

In summary, the fractal descriptors were organized into a feature vector with 116 descriptors: 20 fractal dimensions; 20 lacunarities; 20 average numbers of clusters present in each box; 20 average ratios covered by the largest cluster on each box; 20 percentages of boxes percolated; and 16 curve descriptors, 4 for each of the functions L(r), C(r), Q(r) and P(r).

#### 2.3.2. Deep-Learned Features

In this study, the deep-learned features were extracted from five CNN architectures: DenseNet-121 [58], EfficientNet-b2 [65], Inception-V3 [59], ResNet-50 [60] and VGG-19 [61]. The results were five vectors of deep-learned features. It is important to highlight that in general, the values in the initial layers of a CNN define the quantification of local patterns, such as shape, edge and color. The deeper layers are useful for identifying global patterns, such as texture and semantics [74]. Therefore, in order to explore the global patterns, the normalization layer after the last dense block was chosen from the DenseNet-121 architecture. This layer provided 1024 values. From the EfficientNet-b2, Inception-V3 and ResNet-50 architectures, each final average pooling layer contributed 1408, 2048 and 2048 values, respectively. From VGG-19, the extraction occurred in the last fully connected layer before the output, providing a vector with 4096 values.

### 2.4. Step 3—Feature Ensemble

This step was defined to organize the handcrafted and deep-learned descriptors with the corresponding ensembles, concatenating the features in order to analyze their discriminative capabilities in each of the H&E datasets [84]. It is important to emphasize that each representation obtained through xAI techniques was quantified with fractal approaches (Section 2.3.1), completing the handcrafted set. For each of the five CNN architectures, two feature vectors of xAI representations were defined via fractal techniques, with one from the Grad-CAM images and the other from the LIME images. Each vector considered 116 descriptors (described in Section 2.3.1): 20 fractal dimensions; 20 lacunarity values; 20 average numbers of clusters present in each box; 20 average ratios covered by the largest cluster on each box; 20 percentages of boxes that percolated; and 16 curve descriptors, 4 for each of the functions L(r), C(r), Q(r) and P(r).

In this context, the descriptors explored here were organized into 55 distinct compositions, with 16 individual compositions according to their origins and the number of available descriptors. Among the vectors, three groups of distinct origins were defined: handcrafted, deep-learned and xAI. The handcrafted vectors were obtained through the fractal techniques (*F*), indicating 116 descriptors. The deep-learned vectors were obtained via DenseNet-121 (*D*) with 1024 features, EfficientNet-b2 (*E*) with 1408 features, Inception-V3 (*I*) with 2048 features, ResNet-50 (*R*) with 2048 features and VGG-19 (*V*) with 4096 features. Finally, the xAI vectors were composed of handcrafted descriptors obtained through the application of fractal techniques. In this case, the xAI vectors were defined with 116 descriptors, organized into two groups: those resulting from Grad-CAM explanations, with Grad-CAM DenseNet-121 ($D_{CAM}$), Grad-CAM EfficientNet-b2 ($E_{CAM}$), Grad-CAM Inception-V3 ($I_{CAM}$), Grad-CAM ResNet-50 ($R_{CAM}$) and Grad-CAM VGG-19 ($V_{CAM}$); and those resulting from the LIME explanations, with LIME DenseNet-121 ($D_{LIME}$), LIME EfficientNet-b2 ($E_{LIME}$), LIME Inception-V3 ($I_{LIME}$), LIME ResNet-50 ($R_{LIME}$) and LIME VGG-19 ($V_{LIME}$). The 16 vector compositions with their origins and descriptor numbers are indicated in Table 3.

**Figure 5:**
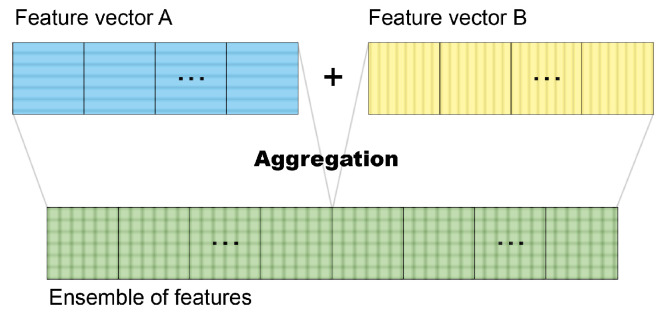
Illustration of the ensemble of features by aggregation.

The remaining 39 feature vectors were created with ensembles of these individual vectors by aggregation, as illustrated in Figure 5. The ensembles were divided into three groups: an ensemble of handcrafted and deep-learned features; an ensemble of deep-learned features; and an ensemble of xAI features. The first group was composed of six vectors, five of them associating the fractal vector with each of the deep-learned features from a CNN, and the other was composed via fractal vector with all the deep-learned features from all CNN models, as presented in Table 4. The ensemble group of deep-learned features was composed of 11 vectors, with 10 permutations of deep-learned features from 2 distinct architectures and one with all available deep-learned features, as shown in Table 5. The last group contained 22 vectors with ensembles of xAI representation descriptors, organized according to the methodology applied to deep-learned ensembles (see Table 6).

### 2.5. Step 4—Feature Selection

The results achieved in Step 3 were vectors with high dimensions, which can lead to classifications with overfitting [43]. In order to avoid this problem, the solution was to apply a dimensionality reduction based on the ranking of the ReliefF technique [85]. This technique was considered to control the number of descriptors under analysis and due to its success found in other studies [45,47,86]. It is important to highlight that the analyzed vectors had dimensions between 116 and 10,740. In this context, considering that the total samples available in our experiments ranged from 58 to 265 images (Table 2), the tests were carried out with totals between 5 and 25 descriptors, starting the analysis process with the maximum value of descriptors and exploring decrements of 5 descriptors, until reaching the smallest dimension [83].

### 2.6. Step 5—Classifier Ensemble and Evaluation Metrics

The last step consisted of carrying out the classifications based on the vectors with an ensemble of heterogeneous classifiers, representative of different categories: support vector machine (SVM) [87], based on functions; naive bayes [88], based on probabilities; random forest [89], based on decision trees; and instance-based K-nearest neighbors [90]. The classifications were combined based on the average of probabilities. In this strategy, the average of all probabilities resulting from each of the classifiers was calculated, so the class with the highest average was selected as the answer (assigned class).

The results were analyzed considering the accuracy metric capable of indicating the global performance of the model (among all the classifications, how many the model classified correctly) [91]. Accuracy was determined through Equation (14), where true positives (TP) are the positive values that the model correctly classified as positive; true negatives (TN) are the negative values that the model correctly classified as negative; false positives (FP) are the negative values that the model incorrectly classified as positive; and false negatives (FN) are the positive values that the model incorrectly classified as negative.

(14) $$Accuracy=\frac{TP+VN}{TP+FP+TN+FN}.$$

**Figure 6:**
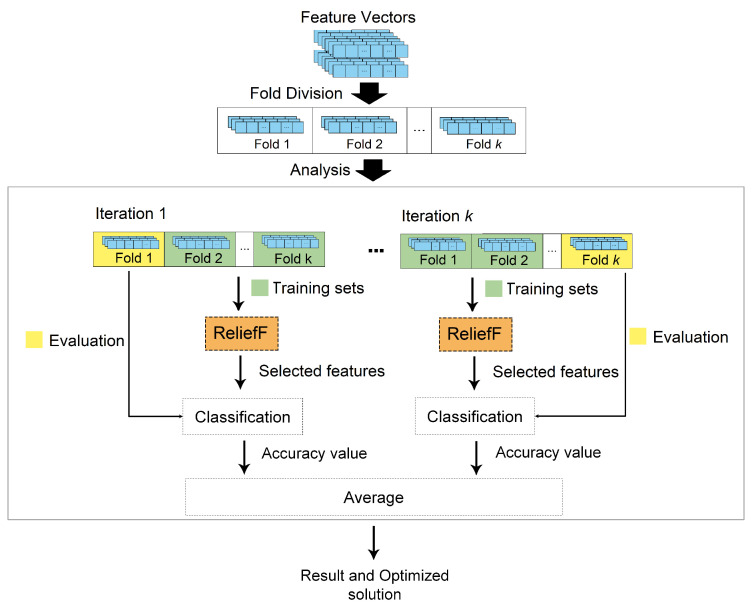
Illustration of the k-fold cross-validation strategy applied in this step.

In addition, the classification process also considered the k-fold cross-validation approach, with k=10. It is important to highlight that the descriptor selection process was applied to each training set of each fold. The selected descriptors were used to perform the classification on the corresponding test set. This technique was applied to reduce the possibility of overfitting occurring, since the result was obtained through the classification average [92]. Figure 6 illustrates this cross-validation process with the selection of descriptors.

### 2.7. Software Packages and Execution Environment

In the proposed scheme, the Pytorch 1.9.0 [71] machine learning library was used to define the convolutional network models. Grad-CAM explanations were obtained via the pytorch-grad-cam 1.3.1 library [93]. The LIME explanations were obtained using the lime 0.2.0.1 library [94], implemented in python [41]. The application of each model was carried out in a remote environment on Google Colab, which provided a free console for executing codes in Python3 and a GPU for processing with 12 GB of available memory. The fractal descriptors were implemented and executed in parts in Matlab R2019b [95] and Python3. Feature selection and classification were processes carried out using the Weka v3.8.5 platform [96]. For this last part, the executions were carried out using an Intel® Core™ i5-8265U 1.60 GHz processor and 8 GB of RAM (Intel, Santa Clara, CA, USA).

## 3. Results and Discussion

The proposed scheme was tested on datasets of histological images (Section 2.1), with comparisons of benign versus malignant (UCSB and CR datasets), healthy versus severe (OED dataset), and male versus female (LG dataset). A performance overview is presented in Section 3.2, with results through the main associations against those from CNNs applied directly to classify the samples and from studies available in the specialized literature.

### 3.1. Feature Ensemble Performance

The combinations were tested according to details presented in Section 2.4, and the 10 highest accuracy values using the smallest number of descriptors were computed to define the average performance in each category. The average performance values are in Table 7 with the highest values in bold.

From the average values, it is noted that the ensemble of deep-learned features provided the highest values in all datasets, with an emphasis on the CR dataset (average accuracy of 100%). In the other datasets, this type of solution indicated accuracy values of 99.66% (LG), 97.23% (OED) and 92.93% (UCSB). Moreover, these facts confirm the feasibility of using the transfer learning strategy in order to obtain relevant compositions for the analysis of histological images. In addition, the ensemble of handcrafted with deep-learned features was the second association capable of indicating relevant accuracy values for the CR (99.76%), LG (99.02%) and OED (96.49%) datasets. Classifications using descriptors based on the xAI representations presented less expressive results, with average accuracy values between 78% and 89% (approximately), but with clear indications about the discriminative potential that can be explored on other research fronts. These facts are relevant contributions to support solutions without overfitting and more robust baseline schemes commonly explored for the improvement of CAD systems focused on H&E images.

These results were analyzed with the Friedman test in order to verify whether there were statistically significant differences between the compositions. The *p*-value was contrasted against α=0.05. Thus, if *p*-value <α, the difference was considered significant. The test was based on the averages of the first 10 results present in each descriptor category. Thus, taking into account a comparison among the six types of feature vectors, the *p*-value was 0.0033, indicating statistically significant differences, with an emphasis on the ensemble of deep-learned features against handcrafted, xAI and xAI ensemble. Each pairwise *p*-value is displayed in Table 8. The differences between the obtained results via an ensemble of deep-learned features and those from an ensemble of handcrafted with deep-learned features were not statistically significant. In addition, the Friedman test provided an average ranking between the performance values of the main associations tested here. The best-ranked combination was the ensemble of deep-learned features, with average accuracy values ranging from 92.93% (UCSB) to 100% (CR).

#### 3.1.1. Details of the Top 10 Solutions

The best combination was defined based on the highest accuracy value using the smallest number of descriptors. Considering this criterion, the obtained rankings are displayed in Table 9, Table 10, Table 11 and Table 12 with the first 10 solutions for the CR, LG, OED and UCSB datasets, respectively. It is noted that the average ranking of the solution validates its position.

In relation to the data collected from the CR dataset (Table 9), it is possible to observe that the 10 best results indicated an accuracy of 100%. It is noted that the descriptors from the DenseNet-121 (D) and EfficientNet-b2 (E) models were those that contributed the most to these results. In these cases, the vectors were defined with a maximum of 20 descriptors, minimizing overfitting.

When the LG dataset was considered (Table 10), it was observed that only one combination indicated an accuracy value of 100%: the ensemble of deep-learned features from the DenseNet-121 (D) and ResNet-50 (R), exploring only 25 descriptors. The contribution of descriptors from the DenseNet-121 was a highlight, present in all vectors of the best results.

For the OED dataset (see Table 11), the highest accuracy was 97.97% with an ensemble of descriptors from the Inception-V3 (I) and VGG-19 (V) architectures, using a reduced number of attributes (20 features). Another combination that achieved the same performance considered the descriptors from the EfficientNet-b2 (E) and Inception-V3 (I) models. However, this last combination involved 25 attributes. In addition, Inception-V3 (I) was the architecture that contributed the most to the best results, followed by DenseNet-121 (D). The main vectors were also defined with up to 25 descriptors.

Finally, considering the UCSB dataset, which is shown in Table 12, it is noted that the highest accuracy value was 94.83%. This performance was defined via the association of deep-learned features from DenseNet-121 (D) with EfficientNet-b2 (E) exploring a total of 25 descriptors. From the top 10 solutions, it is observed that nine compositions were defined based on the DenseNet-121 or ResNet-50 architectures. The total number of descriptors present in the vectors follows the previously found pattern consisting of solutions with a maximum of 25 attributes.

#### 3.1.2. Feature Summary

**Figure 7:**
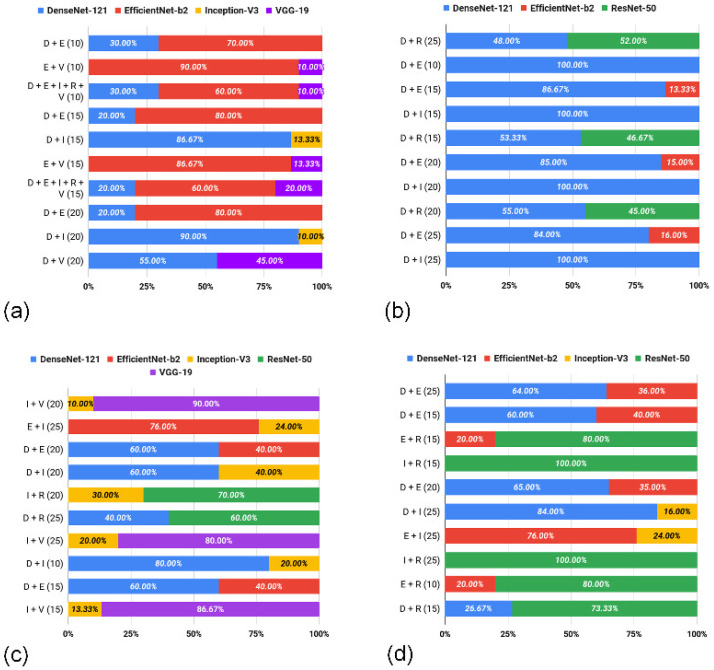
Proportion of features from each CNN in the ensembles, observing the 10 best accuracy values for the CR (**a**), LG (**b**), OED (**c**) and UCSB (**d**) datasets.

The best result was defined through the ensembles of deep-learned features, considering the top 10 solutions for each H&E dataset. For the CR dataset (Figure 7a), it is observed that the compositions were defined mainly based on the EfficientNet-b2 (E) model, representing from 60% to 90% of the features present in 7 of the top 10 solutions. Regarding the LG dataset (Figure 7b), the obtained vectors from the DenseNet-121 (D) architecture predominated (9 of the 10 compositions), indicating from 48% to 100% of the total features in each ensemble. When the OED dataset (Figure 7c) was considered, the highest frequency was obtained through Inception-V3 (I), present in 7 of the 10 compositions, but with a smaller presence of features in the ensembles (from 10% to 40%). Finally, the compositions for the UCSB dataset (Figure 7d) were based on more homogeneous compositions among DenseNet-121 (D), EfficientNet-b2 (E) and ResNet-50 (R). However, in each ensemble, there was a higher incidence of features from the ResNet-50 (R) architecture (from 73% to 100% of the total descriptors).

Based on the details presented previously, some patterns and/or behaviors were observed. The ensembles explored here effectively contributed to a distinction between the H&E images, surpassing the results provided via single-source descriptors. The ensemble of deep-learned features supported the main solutions, with highlights, in terms of occurrence, for the descriptors extracted from the DenseNet-121 (present in 28 of the 40 vectors) and EfficientNet-b2 (present in 19 of the 40 vectors) models. Finally, considering the dimensionalities of the feature vectors, compositions involving 10 to 25 deep-learned features were sufficient to determine the top 10 solutions.

### 3.2. Proposed Scheme versus Fine-Tuned CNN Classifications

**Figure 8:**
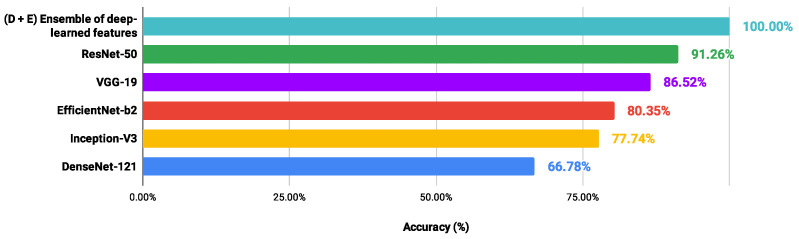
Ranking of accuracy values (%) provided by different approaches in classifying the CR dataset.

**Figure 9:**
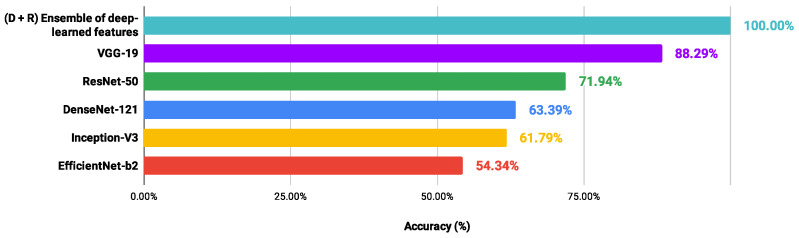
Ranking of accuracy values (%) provided by different approaches in classifying the LG dataset.

**Figure 10:**
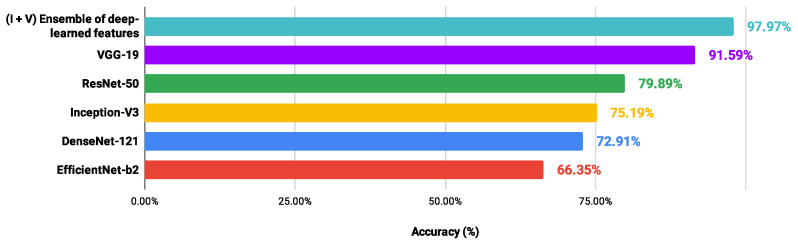
Ranking of accuracy values (%) provided by different approaches in classifying the OED dataset.

**Figure 11:**
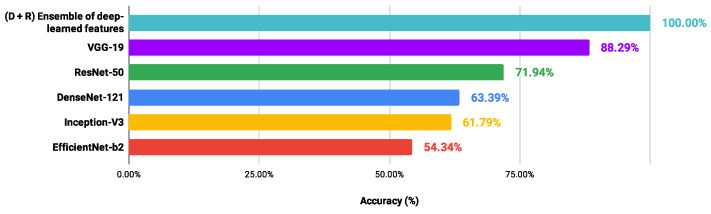
Ranking of accuracy values (%) provided by different approaches in classifying the UCSB dataset.

In order to verify possible gains of the proposed scheme in relation to CNN models applied directly to each H&E dataset, the performance values of the architectures (DenseNet-121 [58], EfficientNet-b2 [65], Inception -V3 [59], ResNet-50 [60] and VGG-19 [61]) were collected after the fine-tuning process. The methodological details were presented in Section 2.2. In addition, Figure 8, Figure 9, Figure 10 and Figure 11 show the best accuracy rates via CNNs against those of the proposed scheme. It is important to highlight that other experiments could be defined, involving different conditions and limits, but those carried out here provided sufficient information to support the contributions of our investigation.

When image classifications were given directly by the CNN architectures, the DenseNet-121 and EfficientNet-b2 models indicated the lowest performance values, ranging from 67.81% to 79.26% and from 54.35% to 80.35%, respectively. The VGG-19 and ResNet-50 networks provided the best performance: LG (88.29%) and OED (97.59%) classified via VGG-19; CR (91.26%) and UCSB (88.52%) through the ResNet-50. However, these accuracy values were lower than those achieved through the proposed scheme, exploring an ensemble of deep-learned features with four heterogeneous classifiers. Also, through the information detailed here, it was noted that the classification performance via a CNN model was not directly related to that achieved through deep-learned features used in an independent process. Most of the best results explored features from the DenseNet-121 and EfficientNet-b2 networks, indicating the lowest performance. These results confirm the contributions obtained in this study.

### 3.3. Performance Overview in Relation to the Literature

Different techniques are available in the specialized literature in order to investigate patterns in histological images, such as for the CR, LG, OED and UCSB datasets [12,13,19,55,69,97,98,99,100]. Therefore, an illustrative overview is important to show the quality of our proposal, indicated in Table 13, Table 14, Table 15 and Table 16 for each of the datasets.

From this illustrative overview, it is possible to conclude that the proposed scheme provided solutions that surpassed a single type of descriptor or even other relevant associations [19,55,69,97,99,100,106,112]. Moreover, the computational scheme was capable of indicating optimized ensembles with the best results for classifying colorectal cancer, liver tissue and oral dysplasia, considering a maximum of 25 descriptors. Finally, these solutions without overfitting and with more robust baseline schemes are for improving CAD systems focused on H&E images. These contributions complement the proposal presented here in this illustrative overview.

## 4. Conclusions

In this work, a computational scheme was developed in order to define the main ensembles of descriptors for the study of histological images, exploring their ranking based on the ReliefF algorithm with a robust ensemble of classifiers (four heterogeneous algorithms). The handcrafted descriptors were established from multiscale and multidimensional fractal techniques (fractal dimension, lacunarity and percolation) and applied to quantify H&E images and their Grad-CAM and LIME representations. The deep-learned features were obtained from multiple CNN architectures, considering the transfer learning strategy. The experiments were carried out on H&E images, representative of breast cancer, colorectal cancer, oral dysplasia and liver tissue.

From the results, the ensemble of deep-learned features provided the highest values in all datasets, with accuracy rates of 94.83% (UCSB), 97.97% (OED) and 100% (CR and LG), exploring a reduced number of features (up to 25 attributes). The descriptors were mainly obtained from the DenseNet-121 and EfficientNet-b2 architectures. In addition, the proposed scheme also indicated that handcrafted ensembles with deep-learned features provided expressive distinctions in the contexts of multiple histological images, with accuracy rates of 99.76% (CR), 99.02% (LG) and 96.49% (OED). This type of composition indicated better performance values than those achieved through individualized analyses, commonly observed in the specialized literature, whether exploring only deep learning [102,103,106,112] or handcrafted techniques [33,69,74,107]. In both categories of ensembles, this study provided useful details and conditions for the community interested in the development and improvement of models for classifying patterns in H&E samples. In relation to the experiments exploring xAI representations, the results were less expressive, with average accuracy values ranging from 78% to 89%. On the other hand, this type of composition achieved results similar to those of directly applied networks, responsible for providing the xAI representations. Thus, we believe that there are still several study avenues to understand the full information capacity present in this type of representation, seeking to improve CAD system designs focused on histological images.

The best solutions were analyzed in relation to the results obtained from consolidated machine-learning techniques, directly applying CNN models to classify the histological datasets. This process considered the DenseNet-121, EfficientNet-b2, Inception-V3, VGG-19 and ResNet-50 architectures. The results were accuracy values from 54.35 to 97.59. The VGG-19 and ResNet-50 networks indicated the best rates: LG (88.29%) and OED (97.59%) via VGG-19; CR (91.26%) and UCSB (88.52%) through ResNet-50. These performance values were lower than those achieved through the best solutions with the proposed scheme. When an illustrative overview was considered in relation to the specialized literature, it was possible to conclude that the proposed scheme provided solutions that surpassed a single type of descriptor or even other relevant associations [55,69,97,106,112]. Moreover, the computational scheme was capable of indicating optimized ensembles with the best results for classifying colorectal cancer, liver tissue and oral dysplasia. Therefore, these conditions highlight the ability of the proposed scheme to present solutions without overfitting and a more robust baseline scheme, with the necessary details for the analysis and testing of CAD systems, focused on H&E samples. Regarding classifications involving representative samples of breast cancer (UCSB dataset), the proposed scheme provided a lower performance, indicating a possible limit of the main solution in this context.

In future works, it is intended to (1) expand the number of handcrafted techniques to quantify H&E images and their representations, especially to define the possible limits involving combinations via xAI; (2) carry out new tests after applying adjustments to the parameters of the CNN architectures in order to verify their impacts on the xAI representations and corresponding quantification; (3) explore multiview learning approaches to complement multiple representations, including investigations into possible gains after applying learning enrichment strategies with fractal techniques.

## Figures and Tables

**Table 1:** Main details of the H&E-stained histological datasets.

Dataset	Image Type	Number of Classes	Classes	Number of Samples	Resolution
UCSB [66]	Breast cancer	2	Malignant and benign	58 (32/26)	896×768
CR [67]	Colorectal cancer	2	Malignant and benign	165 (74/91)	from 567×430 to 775×552
LG [68]	Liver tissue	2	Male and female	265 (150/115)	417×312
OED [69]	Oral epithelial dysplasia	2	Healthy and severe	148 (74/74)	450×250

**Table 2:** Details of the pretrained architectures and respective accuracies (data from [71]) explored to define the deep-learned features and xAI representations.

Architecture	Parameter	Layers	Accuracy (ImageNet)
DenseNet-121 [58]	$8\times 10^{6}$	121	91.97%
EfficientNet-b2 [65]	$9.1\times 10^{6}$	324	95.31%
Inception-V3 [59]	$2.7\times 10^{7}$	48	93.45%
ResNet-50 [60]	$2.6\times 10^{7}$	50	92.86%
VGG-19 [61]	$1.4\times 10^{8}$	19	90.87%

**Table 3:** Feature vectors obtained via different techniques.

Origin	Composition	Number of Features
Handcrafted	Fractals (F)	116
Deep learned	DenseNet-121 (D)	1024
	EfficientNet-b2 (E)	1408
	Inception-V3 (I)	2048
	ResNet-50 (R)	2048
	VGG-19 (V)	4096
xAI	Grad-CAM DenseNet-121 ($D_{CAM}$)	116
	Grad-CAM EfficientNet-b2 ($E_{CAM}$)	116
	Grad-CAM Inception-V3 ($I_{CAM}$)	116
	Grad-CAM ResNet-50 ($R_{CAM}$)	116
	Grad-CAM VGG-19 ($V_{CAM}$)	116
	LIME DenseNet-121 ($D_{LIME}$)	116
	LIME EfficientNet-b2 ($E_{LIME}$)	116
	LIME Inception-V3 ($I_{LIME}$)	116
	LIME ResNet-50 ($R_{LIME}$)	116
	LIME VGG-19 ($V_{LIME}$)	116

**Table 4:** Ensembles of handcrafted and deep-learned features.

Composition	Number of Features
F + D	1140
F + E	1524
F + I	2164
F + R	2164
F + V	4212
F + D + E + I + R + V	10,740

**Table 5:** Ensembles of deep-learned features.

Composition	Number of Features
D + E	2432
D + I	3072
D + R	3072
D + V	5120
E + I	3456
E + R	3456
E + V	5504
I + R	4096
I + V	6144
R + V	6144
D + E + I + R + V	10,624

**Table 6:** Ensembles of xAI features.

Composition	Number of Features
$D_{CAM}$ + $E_{CAM}$	232
$D_{CAM}$ + $I_{CAM}$	232
$D_{CAM}$ + $R_{CAM}$	232
$D_{CAM}$ + $V_{CAM}$	232
$E_{CAM}$ + $I_{CAM}$	232
$E_{CAM}$ + $R_{CAM}$	232
$E_{CAM}$ + $V_{CAM}$	232
$I_{CAM}$ + $R_{CAM}$	232
$I_{CAM}$ + $V_{CAM}$	232
$R_{CAM}$ + $V_{CAM}$	232
$D_{CAM}$ + $E_{CAM}$ + $I_{CAM}$ + $R_{CAM}$ + $V_{CAM}$	580
$D_{LIME}$ + $E_{LIME}$	232
$D_{LIME}$ + $I_{LIME}$	232
$D_{LIME}$ + $R_{LIME}$	232
$D_{LIME}$ + $V_{LIME}$	232
$E_{LIME}$ + $I_{LIME}$	232
$E_{LIME}$ + $R_{LIME}$	232
$E_{LIME}$ + $V_{LIME}$	232
$I_{LIME}$ + $R_{LIME}$	232
$I_{LIME}$ + $V_{LIME}$	232
$R_{LIME}$ + $V_{LIME}$	232
$D_{LIME}$ + $E_{LIME}$ + $I_{LIME}$ + $R_{LIME}$ + $V_{LIME}$	580

**Table 7:** Average accuracy values (%) computed from the 10 best results in each combination and for each type of H&E image.

	CR	LG	OED	UCSB
**Handcrafted**	84.48% ± 2.05	90.42% ± 5.23	87.03% ± 1.78	72.41% ± 1.89
**Deep learned**	99.27% ± 0.76	98.49% ± 0.65	96.28% ± 0.55	91.38% ± 1.54
**xAI**	83.82% ± 0.90	86.98% ± 2.54	80.88% ± 0.43	78.28% ± 1.14
**Ensemble of handcrafted ** **and deep learned**	99.76% ± 0.30	99.02% ± 0.51	96.49% ± 0.66	90.52% ± 0.86
**Ensemble of deep learned**	**100%**	**99.66% ± 0.11**	**97.23% ± 0.47**	**92.93% ± 0.93**
**Ensemble of xAI**	86.18% ± 1.45	89.62% ± 0.42	78.85% ± 0.61	78.45% ± 1.16

**Table 8:** Ranking of the best associations with their respective pairwise *p*-values, according to average accuracy values and the Friedman test.

*p*-Value	Ensemble of Deep Learned	Ensemble of Handcrafted and Deep Learned	Deep Learned	Handcrafted	Ensemble of xAI	xAI	Average Ranking
**Ensemble of deep learned**	-	0.3955	0.2396	0.0191	0.0191	0.0066	1
**Ensemble of handcrafted** **and deep learned**	0.3955	-	0.7313	0.1006	0.1006	0.0380	2.25
**Deep learned**	0.2396	0.7313	-	0.1819	0.1819	0.0735	2.75
**Handcrafted**	0.0191	0.1006	0.1819	-	1.0000	0.6073	4.75
**Ensemble of xAI**	0.0191	0.1006	0.1819	1.0000	-	0.6073	4.75
**xAI**	0.0066	0.0380	0.0735	0.6073	0.6073	-	5.5

**Table 9:** Top 10 results for the classification of the CR dataset with feature vectors composed of ensembles of deep-learned features.

Feature Vector	Size	Accuracy (%)	F-1 Score
D + E	10	100	1.000
E + V	10	100	1.000
D + E + I + R + V	10	100	1.000
D + E	15	100	1.000
D + I	15	100	1.000
E + V	15	100	1.000
D + E + I + R + V	15	100	1.000
D + E	20	100	1.000
D + I	20	100	1.000
D + V	20	100	1.000

**Table 10:** LG dataset: top 10 results exploring the ensembles of deep-learned features.

Feature Vector	Size	Accuracy (%)	F-1 Score
D + R	25	100	1.000
D + E	10	99.62	0.996
D + E	15	99.62	0.996
D + I	15	99.62	0.996
D + R	15	99.62	0.996
D + E	20	99.62	0.996
D + I	20	99.62	0.996
D + R	20	99.62	0.996
D + E	25	99.62	0.996
D + I	25	99.62	0.996

**Table 11:** OED dataset: top 10 results via the ensembles of deep-learned features.

Feature Vector	Size	Accuracy (%)	F1-Score
I + V	20	97.97	0.980
E + I	25	97.97	0.980
D + E	20	97.30	0.973
D + I	20	97.30	0.973
I + R	20	97.30	0.973
D + R	25	97.30	0.973
I + V	25	97.30	0.973
D + I	10	96.62	0.966
D + E	15	96.62	0.966
I + V	15	96.62	0.966

**Table 12:** UCSB dataset: Top 10 results exploring the ensembles of deep-learned features.

Feature Vector	Size	Accuracy (%)	F1-Score
D + E	25	94.83	0.948
D + E	15	93.10	0.931
E + R	15	93.10	0.931
I + R	15	93.10	0.931
D + E	20	93.10	0.931
D + I	25	93.10	0.931
E + I	25	93.10	0.931
I + R	25	93.10	0.931
E + R	10	91.38	0.914
D + R	15	91.38	0.914

**Table 13:** Classification of colorectal samples: accuracy values (%) provided by different approaches.

Method	Approach	Accuracy (%)
Proposed	DenseNet-121 and EfficientNet-b2 (ensemble of deep-learned features)	100%
Roberto et al. [12]	ResNet-50, fractal dimension, lacunarity and percolation (ensemble of handcrafted and deep-learned features)	99.39%
Dabass et al. [101]	31-layer CNN (deep learning)	96.97%
de Oliveira et al. [19]	ResNet50 (activation_48_relu layer), ReliefF and 35 deep-learned features	98.00%
Tavolara et al. [97]	GAN and U-Net (deep learning)	94.02%
Sena et al. [102]	12-layer CNN (deep learning)	93.28%
Segato dos Santos et al. [81]	Sample entropy and fuzzy logic (handcrafted)	91.39%
Roberto et al. [33]	Percolation (handcrafted)	90.90%
Bentaieb and Hamarneh [103]	U-Net and AlexNet (deep learning)	87.50%
Zhang et al. [99]	ResNet deep-tuning (DL)	86.67%
Awan et al. [104]	Color normalization, U-Net and GoogLeNet (deep learning)	85.00%

**Table 14:** Classification of liver tissue: accuracy values (%) via different approaches.

Method	Approach	Accuracy (%)
Proposed	DenseNet-121 and ResNet-50 (ensemble of deep-learned features)	100%
Di Ruberto et al. [105]	Statistical analysis and texture features (handcrafted)	100%
Nanni et al. [13]	6 CNNs and handcrafted features (ensemble of handcrafted and deep-learned features)	100%
Roberto et al. [12]	ResNet-50, fractal dimension, lacunarity and percolation (ensemble of handcrafted and deep-learned features)	99.62%
de Oliveira et al. [19]	ResNet50 (activation_48_relu layer), ReliefF and 5 deep-learned features	99.32%
Andrearczyk and Whelan [106]	Texture CNN (deep learning)	99.10%
Watanabe et al. [107]	GIST descriptor, PCA and LDA (handcrafted)	93.70%

**Table 15:** Classification of oral dysplasia: accuracy rates (%) provided by different methods.

Method	Approach	Accuracy (%)
Proposed	Inception-V3 and VGG-19 (ensemble of deep-learned features)	97.97%
Adel et al. [108]	SIFT, SURF, ORB (handcrafted)	92.80%
Azarmehr et al. [100]	Neural architecture search and handcrafted descriptors (morphological and nonmorphological)	95.20%
Silva et al. [69]	Morphological and nonmorphological features (handcrafted)	92.40%
Maia et al. [57]	Densenet121	91.91%
Krishnan et al. [109]	Fractal dimension, wavelet, Brownian movement and Gabor filters (handcrafted)	88.38%

**Table 16:** Classification of breast cancer: accuracy values (%) trough different strategies.

Method	Approach	Accuracy (%)
Li et al. [110]	RefineNet and Atrous DenseNet (deep learning)	97.63%
Yu et al. [98]	CNN, LBP, SURF, GLCM and other handcrafted features (ensemble handcrafted and deep-learned features)	96.67%
Proposed	DenseNet-121 and EfficientNet-b2 (ensemble of deep-learned features)	94.83%
Feng et al. [111]	Stacked denoising autoencoder (deep learning)	94.41%
Kausar et al. [55]	Color normalization, Haar wavelet decomposition and 16-layer CNN (deep learning)	91.00%
Roberto et al. [12]	ResNet-50, fractal dimension, lacunarity and percolation (ensemble of handcrafted and deep-learned features)	89.66%
Roberto et al. [33]	Percolation (handcrafted)	86.20%
Papastergiou et al. [112]	Spacial decomposition and tensors (deep learning)	84.67%
Araújo et al. [113]	Color normalization, 13-layer CNN and SVM (deep learning)	83.30%

## Data Availability

Data are contained within the article.

## Abbreviations

The following abbreviations are used in this manuscript:

H&E	Hematoxylin and eosin
CAD	Computer-aided diagnosis
CNN	Convolutional neural network
xAI	Explainable artificial intelligence
CAM	Class activation mapping
Grad-CAM	Gradient-weighted class activation mapping
LIME	Local interpretable model-agnostic explanations
UCSB	Breast cancer dataset
CR	Colorectal cancer dataset
LG	Liver tissue dataset
OED	Oral epithelial dysplasia dataset
SGDM	Stochastic gradient descent
F	Fractal techniques
D	DenseNet-121
E	EfficientNet-b2
I	Inception-V3
R	ResNet-50
V	VGG-19
$D_{CAM}$	Grad-CAM representation via DenseNet-121
$E_{CAM}$	Grad-CAM representation via EfficientNet-b2
$R_{CAM}$	Grad-CAM representation via ResNet-50
$I_{CAM}$	Grad-CAM representation via Inception-V3
$V_{CAM}$	Grad-CAM representation via VGG-19
$D_{LIME}$	LIME representation via DenseNet-121
$E_{LIME}$	LIME representation via EfficientNet-b2
$I_{LIME}$	LIME representation via Inception-V3
$R_{LIME}$	LIME representation via ResNet-50
$V_{LIME}$	LIME representation via VGG-19
VP	True positives
VN	True negatives
FP	False positives
FN	False negatives
